# Rupture of totally implantable central venous access devices (Intraports) in patients with cancer: report of four cases

**DOI:** 10.1186/1477-7819-2-36

**Published:** 2004-10-19

**Authors:** Dimitrios K Filippou, Christoforos Tsikkinis, Georgios K Filippou, Athanasios Nissiotis, Spiros Rizos

**Affiliations:** 1 Dept. of Surgical Oncology, Agii Anargiri Kifissias Anticancer Hospital, Greece; 2 1^st ^Surgical Department, GPHP "Tzanion" Hospital, Piraeus, Athens, Greece; 3 3^rd ^Surgical Department, Piraeus Anticancer Hospital "Metaxa", Piraeus, Greece

## Abstract

**Background:**

Totally implantable central venous access devices (intraports) are commonly used in cancer patients to administer chemotherapy or parenteral nutrition. Rupture of intraport is a rare complication.

**Patients and methods:**

During 3 years period, a total of 245 intraports were placed in cancer patients for chemotherapy. Four of these cases (two colon cancer and one each of pancreas and breast cancer) had rupture of the intraport catheter, these forms the basis of present report.

**Results:**

Mean time *insitu *for intraports was 164∀35 days. Median follow-up time was 290 days and total port time *in situ *was 40180 days. The incidence of port rupture was 1 per 10,000 port days.

Three of the 4 cases were managed by successful removal of catheters. In two of these the catheter was removed under fluoroscopic control using femoral route, while in the third patient the catheter (partial rupture) was removed surgically. One of the catheters could not be removed and migrated to right ventricle on manipulations.

**Conclusion:**

Port catheter rupture is a rare but dreaded complication associated with subcutaneous port catheter device placement for chemotherapy. In case of such an event the patient should be managed by an experienced vascular surgeon and interventional radiologist, as in most cases the ruptured catheter can be retrieved by non operative interventional measures.

## Background

Totally implantable central venous access devices (intraports) are commonly used in patients with cancer to administer chemotherapy, blood and blood products, antibiotics, parenteral nutrition and to obtain blood samples for laboratory analysis. The catheter is usually placed in the subclavian vein under local anesthesia. There are many complications associated with this technique like hemothorax, pneumothorax, pocket infection, infection of the tunnel or the port, bleeding, hematoma and thrombosis of the catheter or the vein. A very rare complication of the intraport catheter is rupture inside the subclavian vein [1].

The literature regarding common complications is abound, however there is very little information on port ruptures. Some attempts has been made to correlate these with type of port used, site of placement, type of chemotherapy, duration of catheter use etc [2,3]. However, what is ideal is still debatable. We report here 4 cases of intraport ruptures encountered in our practice.

## Patients and methods

Between June 1995 and May 1998, 245 patients underwent intraports insertions at the "Agii Anargiri" Anticancer Hospital of Kifissia, Athens, Greece, and were followed up for possible device-related morbidity. Baseline demographic information and the indication for the placement were obtained from the retrospective review of patients' medical and operative reports. Follow-up continued till the device was removed or the patient died. Follow-up time ranged from 1 month to over 3 years. Port-A-Cath^® ^with titanium portal and detachable silicon rubber catheter (Arrow International™, USA) were used in all cases. All patients requiring removal or replacement of the device, prior to completion of the intended treatment were identified from the operative room and hospital records.

All intraports were inserted by percutaneous access technique to the subclavian vein. The tip of the catheter was positioned in the superior vena cava or the right atrium. The ports were then placed within a subcutaneous pocket created on the anterior chest wall. The position was documented by immediate intraoperative chest roentgenogram. Four of these patients had port rupture of which three were complete and one partial, one of these ruptures was accidental.

Routine removal of the subcutanous intraport was carried out in the operating room under local anesthesia with xylocaine 1% (10 cc). An incision was placed on the skin in the area over the drum of the device. Then the drum was prepared by cutting off the tissues that surround the port. The port and the catheter were caught with Kocher forceps. The tissues around the catheter were dissected and the catheter was slowly pulled out. The catheter was cut into three pieces and was sent for culture. Patients were discharged after two hours of observation.

## Results

Over a three year period between 1995 and 1998 a total of 245 port devices were fitted in cancer patients (139 females and 106 males). Mean age of the patients was 58 ± 6.3 years. Total port time *in situ *was 40180 days, while mean (SD) port time *in situ *was 164 (35) days.

After a median follow-up of 290 days (range 30–690 days) four ports ruptured, thus bringing the number of fractures per 1000 port days to 0.01% or one rupture every 10,000 port days. Two patients had cancer of the colon and one each had cancer of breast and pancreas. Both patients with colonic cancer received 5 flurouracil and leukovorin, patient with breast cancer received epirubicin and paclitaxal and patient with pancreatic cancer received gemcitabine, paclitaxal and cisplatinum. No correlation was observed with type of chemotherapy or site of disease. Three of the port ruptures were full and one partial one of the ruptures was accidental (case 1). The detail of the cases has been provided in next section. Three of the patients survived for two years after the catheter removal, while the fourth patient with accidental port rupture and whose catheter was left behind in the right ventricle died of progressive disease two month later.

### Case 1

A 65-years-old female with colonic cancer, received intraport for chemotherapy administration. After 6 months due to the poor response to chemotherapy it was decided to remove the catheter. During the manipulations for removal, the catheter, accidentally ruptured at the point of its entrance in to subclavian vein. The peripheral part of the catheter remained in the vein, while only the central part could be removed. Another attempt to uncover the subclavian vein till superior vena cava failed. Patient underwent thoracotomy for removal of remaining catheter two days later. Superior vena cava was opened and catheter removal was attempted, however, during this process the catheter slipped into right atrium and further attempts to retrieve it were abandoned. Patient was started on anticoagulant treatment using *enoxaparin sodium*, 1 mg/kg/12 h for 5 days and 40 mg/day for further 14 days in order to prevent thromboembolic event. The patient died two months later due to progressive disease without obvious complications related to the retained catheter.

### Case 2

A 68-year-old female with colonic cancer received intraport for administration of chemotherapy. After one and a half year of treatment it was decided to remove the catheter as the catheter has thrombosed due to non heparinization. At the time of its removal the catheter ruptured at the point of its entry to subclavian vein. The peripheral part of the catheter remained in the vein. An unsuccessful attempt was made to expose subclavian vein till superior vena cava. Later this catheter migrated to right ventricle. The catheter was removed using the technique of Yedlicka *et al *[5], through the left femoral vein by advancing a vessel catheter to right ventricle under fluoroscopic control. The broken catheter was caught with endovessel forceps and was removed through femoral vein.

### Case 3

**Figure 1:**
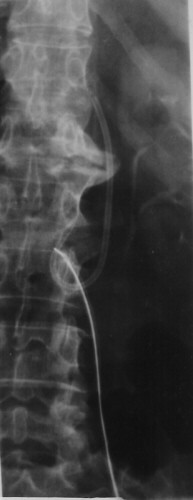
Chest X-ray showing the port in correct position while the catheter is not shown here. The catheter had moved in the right atrium, from where it was removed fluoroscopically.

**Figure 2:**
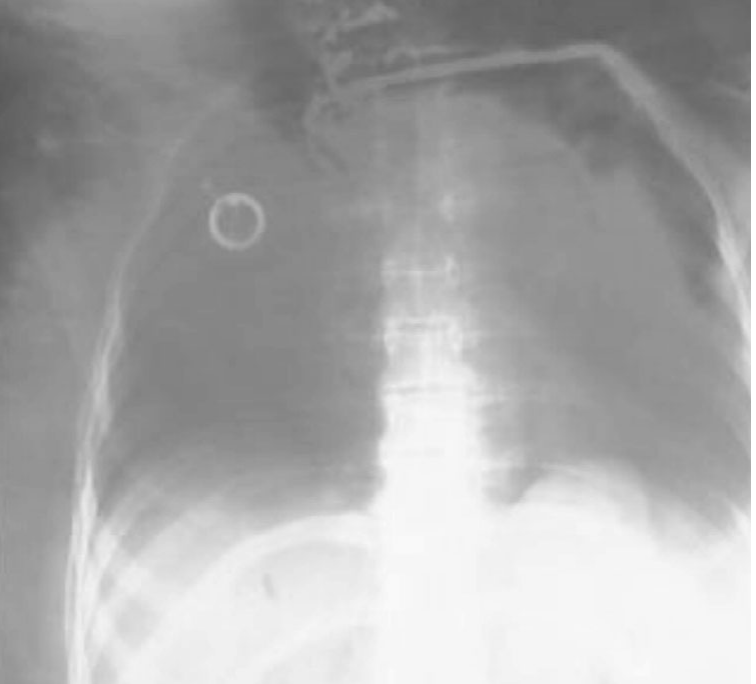
X-ray showing the intravascular catheter being fluoroscopically removed by means of a special endo-vessel grasper.

**Figure 3:**
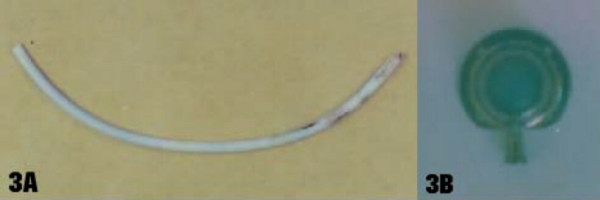
**(a) **The ruptured catheter had been sent for biomechanical analysis that identified alteration of its elastic properties. **(b) **The port removed by means of the open technique

***A ***74-years-old female suffering from breast cancer underwent intraport insertion for administration of chemotherapy. After fourteen months of treatment it was decided to remove the catheter as the catheter had thrombosed. On attempted removal the catheter was found to be ruptured at its entry to subclavian vein (Figure 1). *N*ext day, the broken part of the catheter was removed successfully under fluoroscopic control using the technique described above for case 2 (Figure 2). No complications were observed. The biomechanical analysis of removed catheter showed a significant decrease in the elasticity of the material (Figure 3).

### Case 4

In a 56-year-old female patient with pancreatic carcinoma underwent an intraport placement for chemotherapy. Eight months later, the patient complained of pain in the back during the administration of chemotherapy. A fluoroscopic examination showed partially broken catheter in the vein while the other part was lying in the subcutaneous tissue. The catheter was removed from the subclavian vein carefully to avoid complete rupture of the catheter. Similar to case 3 above, the biomedical examination showed a significant reduction in the elasticity of the catheter material.

## Discussion

Since Aubaniac first described the percutaneous central venous catheterization in 1952, insertion of central venous access devices for fluid administration has increased rapidly. The total complication rate associated with central venous catheter devices ranges from 0.4 to 29% [3,4,6].

Spontaneous rupture of the port catheters appears to be a very rare and dreaded event. Biffi *et al*, in 1997 [1], reported three cases of port catheter rupture out of 178 ports inserted by them. The incidence of port rupture was estimated to be 1.68 % (0.09/1000 port days). In their series the rupture occurred 66 days after the placement, during a pause between subsequent chemotherapy cycles. The symptoms consisted of palpitations and chest discomfort in two while the third patient remained asymptomatic. All the catheters in their series were removed by interventional technique without any complications [1,5].

The biomechanical analysis, of ruptured catheters in our series showed a significant decrease in the elasticity of the material. No correlation between changes of mechanical properties of the material and specific chemotherapy administrated through the port has been established so far [7,8]. Even in our series all patients received differing chemotherapy and no correlation was observed between the agent and property of the material, this is also due to smaller number of such events.

In case 1 the port catheter rupture was due to wrong manipulations during catheter removal and accidental cutting of peripheral part without first holding it in clamp. The further catheter movement was induced by negative intra-thoracic pressure during respiration [9]. Other potential cause of catheter fracture could be incorrect fixation of the catheter to the locking steel ring, repeated high pressure injections to resolve clot formation, alteration of the catheter mechanical properties etc. In two of our cases decreased elasticity of the material was found however one case could not be attributed to any know cause. There was no correlation of port rupture with type of chemotherapy or site of cancer probably due to only 4 events.

Ballarini *et al *[10], suggested that catheter thrombosis mainly occurs due to incorrect fixation of the locking steel ring to the port where it is associated with rupture. The estimated incidence of port catheter rupture and embolization varies from 0.9 to 1.7% of the cases [1,10-12]. It was 1.65% in our series.

There is no controversy that the foreign bodies inserted in the systematic circulation must be removed. This is best achieved under fluoroscopy with specific catheters and snare loops. Removal of intravascular material by means of minimally invasive techniques presents excellent results, while at the same time it minimizes morbidity and mortality [13,14]. If such an attempts fail, open surgery should be considered.

## Conclusions

Intravascular rupture of subcutaneous port catheters is a rare complication. Etiology still remains elusive, however wrong placement has been advocated as the most important cause. Other causes include catheter material faults and alterations of the material's mechanical properties, probably due to the administered substances; however, there is no data to support the effect of administered substances. Ruptured catheters are best removed by minimally invasive radiological techniques.

## Competing interests

The authors declare that they have no competing interests.

## Authors' contributions

**DF **participated in the operations, and drafted the manuscript.

**CT **participated in the operations, patients follow-up search of literature and preparation of draft.

**GF **conducted the follow-up and helped to draft the manuscript.

**AN **and **SR **participated in the operations, design of the study and preparation of manuscript for publication.

All authors read and approved the final manuscript.
